# Trends in mortality with osteoporosis recorded as the underlying cause of death among adults aged 65 years and older in the United States, 1999–2024

**DOI:** 10.3389/fmed.2026.1892346

**Published:** 2026-09-14

**Authors:** Chengzhuan Luo, Qian Zhou

**Affiliations:** 1 Department of Hand Surgery, Linping Hospital, The Second Affiliated Hospital, Zhejiang University School of Medicine, Hangzhou, Zhejiang, China; 2 Department of Emergency, The Second Affiliated Hospital, Zhejiang University School of Medicine, Hangzhou, Zhejiang, China; 3 School of Nursing, Anhui Medical University, Hefei, Anhui, China

**Keywords:** age-adjusted mortality rate, CDC WONDER, mortality, osteoporosis, trend, United States

## Abstract

**Objective:**

Osteoporosis coded as the underlying cause of death increases mortality risk in older adults. This study aimed to examine temporal trends and demographic disparities in mortality with osteoporosis as the underlying cause of death among U.S. adults aged 65 years and older from 1999 to 2024, and to identify recent mortality plateauing patterns.

**Methods:**

A population-based retrospective ecological analysis was performed using the CDC WONDER national mortality database. All eligible decedents were restricted to adults ≥65 years with ICD-10 M80/M81 recorded as the underlying cause of death. Mortality counts, age-adjusted mortality rates (AAMR), crude mortality rates (CMR for age subgroups), and average annual percent change (AAPC) with 95% confidence intervals (CIs) were stratified by sex, census region, race/ethnicity, urbanization (limited to 1999–2020), and age group. Annual cells with death counts <10 were suppressed and excluded from continuous trend plotting. Joinpoint regression with pairwise AAPC *Z*-tests was applied to quantify and compare subgroup trends.

**Results:**

Total deaths with osteoporosis as the underlying cause decreased from 1,622 in 1999 to 484 in 2024 (−70.16%). The overall AAMR declined from 4.78 (95% CI: 4.54–5.01) to 0.92 (95% CI: 0.84–1.00), with an overall AAPC of −6.26 (95% CI: −7.37–−5.14; *P* < 0.05). Statistically significant full-period downward AAPCs were observed across all demographic subgroups. However, segmented APC 95% CIs crossed zero in recent years for most groups, indicating widespread mortality plateauing after long-term decline. Formal pairwise comparisons confirmed faster annual mortality reduction in Southern census regions and metropolitan areas. For Hispanic adults aged ≥65 years, annual osteoporosis-related death counts were consistently less than 10 from 1999 to 2024, resulting in massive suppressed annual data that cannot support a continuous trend curve, though valid aggregate cross-sectional mortality metrics for this subgroup are fully available. Females, individuals aged ≥85 years, and nonmetropolitan residents sustained higher mortality burden.

**Conclusions:**

Mortality with osteoporosis documented as the underlying cause of death presented a significant overall downward trend among U.S. adults aged 65 and older over 1999–2024, yet most subgroups exhibited plateauing mortality in recent calendar years. Continuous annual trend visualization for Hispanic populations is unfeasible due to CDC WONDER privacy suppression rules that hide records with fewer than 10 annual deaths, while complete aggregate summary statistics for Hispanic participants are reported. Females, the oldest-old population, and rural residents carried persistent excess mortality burden. These stratified nationwide epidemiological findings support targeted bone health public interventions for high-risk elderly subgroups

## Introduction

Osteoporosis is a systemic skeletal disorder characterized by low bone mass, microarchitectural deterioration of bone tissue, and enhanced bone fragility, leading to substantially increased risk of fragility fractures including vertebral, hip, and other non-vertebral fractures (1). It represents a major global public health problem associated with considerable disability, reduced quality of life, and elevated mortality risk among affected individuals, especially the elderly aged ≥65 years (2). With population aging and prolonged life expectancy, the population-level burden of osteoporosis and its related adverse outcomes continues to attract widespread attention (3). In the United States, osteoporosis affects a large proportion of older adults and contributes to substantial medical costs and mortality when recorded as the underlying cause of death (4). Although numerous studies have investigated the prevalence, incidence, and fracture-related mortality of osteoporosis, long-term nationwide trends in mortality directly attributable to osteoporosis as an underlying cause remain incompletely characterized (5). Reliable population-based data on mortality trends, age-adjusted mortality rates (AAMR), and annual percentage changes are critical for public health planning, resource allocation, and intervention evaluation (6). The CDC WONDER database provides a valid and comprehensive source for population-based mortality surveillance in the United States (7). A key data limitation of this database is privacy suppression: annual death counts fewer than 10 are hidden from export, which disrupts continuous time-series plotting for small subgroups such as Hispanic older adults. To date, studies documenting long-term mortality trends of osteoporosis as the underlying cause of death over a 26-year period using this database remain limited, particularly those with stratified analyses by sex, census region, race/ethnicity, urbanization, and age groups. The present study was conducted to examine the temporal trends of mortality with osteoporosis as the underlying cause of death in the United States from 1999 to 2024, aiming to provide updated and detailed population-level evidence for bone health policy and clinical practice.

## 2 Methods

### 2.1 Study design and period

A retrospective epidemiological analysis was performed to characterize trends in mortality with osteoporosis as the underlying cause of death in the United States over the 26-year period (1999–2024). All analyses were restricted to decedents aged ≥65 years. This study was exempt from institutional review board approval, as we utilized a de-identified government-provided public-use dataset in accordance with Strengthening the Reporting of Observational Studies in Epidemiology (STROBE) guidelines (8).

### 2.2 Data source and case definition

#### 2.2.1 Database selection

Mortality data were retrieved from the CDC WONDER database. This database aggregates cause-of-death information from death certificates for all 50 U.S. states and the District of Columbia, with established utility in prior analyses of mortality trends due to its long-term coverage, population-level scope, and adherence to standardized data collection protocols (7). A core built-in privacy rule suppresses all annual cells with death counts <10 to protect individual identity.

#### 2.2.2 Case identification

Deaths with osteoporosis as the underlying cause were defined using the International Classification of Diseases, 10th Revision (ICD-10) codes M80 (osteoporosis with pathological fracture) and M81 (osteoporosis without pathological fracture) (9). Only deaths where osteoporosis was documented as the underlying cause of death and the decedent was aged ≥65 years were included. Full CDC WONDER query criteria: underlying cause ICD-10 M80/M81; death year range 1999–2024; age filter ≥65 years; stratification variables including sex, four U.S. census regions, race/ethnicity (Hispanic, NH White, NH Other), NCHS 2013 urban-rural classification, and age groups 65–74, 75–84, ≥85 years. Critical data limitation note: Annual mortality observations for the Hispanic subgroup were frequently suppressed across 1999–2024 due to case counts <10, resulting in discontinuous time-series data unsuitable for continuous trend line plotting. Aggregate cross-sectional 1999 and 2024 mortality values and full-period AAPC calculations remain available and are reported in Table 1, but a complete annual trend curve cannot be generated for visualization. Urban-rural population classification data were only available up to 2020, so urbanization subgroup analyses were limited to 1999–2020. Cells with annual death counts <10 were automatically suppressed by CDC WONDER to protect individual privacy and excluded from trend regression models and continuous figure plotting.

**Table 1:** Mortality with osteoporosis recorded as the underlying cause of death among adults aged ≥65 years in the United States, 1999–2024.

Characteristic	Deaths			AAMR		
	1999	2024	Percent Change (%)	1999 (95% CI)	2024 (95% CI)	AAPC (95% CI)
Total	1,622	484	−70.16	4.78 (4.54–5.01)	0.92 (0.84–1.00)	−6.26 (−7.37–−5.14)*
Sex						
Female	1,466	416	−71.62	6.39 (6.07–6.72)	1.27 (1.15–1.40)	−6.09 (−7.12–−5.04)*
Male	156	68	−56.41	1.37 (1.15–1.59)	0.30 (0.23–0.39)	−6.13 (−7.77–−4.47)*
Census Region						
Northeast	246	71	−71.14	3.33 (2.91–3.75)	0.67 (0.52–0.85)	−5.66 (−7.16–−4.13)*
Midwest	513	150	−70.76	6.06 (5.53–6.58)	1.32 (1.11–1.55)	−5.85 (−7.24–−4.44)*
South	442	138	−68.78	3.78 (3.42–4.13)	0.69 (0.57–0.81)	−7.01 (−8.73–−5.26)*
West	421	125	−70.31	6.45 (5.83–7.07)	1.02 (0.85–1.22)	−6.85 (−7.88–−5.80)*
Race						
Hispanic	28	15	−46.43	2.19 (1.44–3.18)	0.32 (0.18–0.52)	−6.65 (−7.78–−5.84)*
NH White	1,548	442	−71.45	5.22 (4.96–5.49)	1.10 (1.00–1.21)	−6.01 (−6.99–−5.01)*
NH Other	40	27	−32.50	1.25 (0.89–1.71)	0.29 (0.18–0.44)	−5.38 (−9.77–−0.77)*
Age Groups^a^						
65−74 years	70	31	−55.71	0.38 (0.30–0.48)	0.09 (0.06–0.12)	−6.27 (−9.87–−2.54)*
75-84 years	428	110	−74.30	3.50 (3.17–3.83)	0.57 (0.46–0.68)	−7.04 (−8.95–−5.10)*
85 + years	1,124	343	−69.48	27.06 (25.48–28.64)	5.33 (4.77–5.89)	−6.11 (−7.23–−4.98)*

*P < 0.05. ^a^For the age groups, the crude mortality rate was used as a substitute for AAMR, and the AAPC was computed based on the crude mortality rate. AAMR, age-adjusted mortality rate; CI, confidence interval; AAPC, average annual percent change; NH, non-Hispanic.

### 2.3 Data abstraction

Data were extracted from the CDC WONDER database using predefined criteria to capture key variables associated with mortality where osteoporosis was the underlying cause of death: year of death, annual population size, U.S. Census region of residence, urban-rural classification, age at death, sex, and race/ethnicity.

Age at death was stratified into three groups: 65–74 years, 75–84 years, and 85 years and older. Sex was dichotomized as female or male. Race/ethnicity was classified into three mutually exclusive groups: Hispanic, Non-Hispanic (NH) White, and NH Other. Urban-rural classification was determined using the 2013 National Center for Health Statistics (NCHS) scheme (10). U.S. Census regions were defined per the U.S. Census Bureau's standard classifications: Northeast, Midwest, South, and West.

For urbanization subgroup analysis, complete population classification data only extended to 2020; thus all AAMR and AAPC estimates for metropolitan/nonmetropolitan subgroups were restricted to 1999–2020, and no 2024 urban-rural rate metrics were reported. For age subgroups, crude mortality rates (CMR) were used instead of AAMR, and corresponding AAPC values were calculated based on CMR. For racial/ethnic subgroups: continuous annual trend plotting was only feasible for NH White and NH Other due to persistent privacy suppression of Hispanic annual data; full aggregate summary statistics for Hispanic participants are retained in all tabular results.

### 2.4 Statistical analysis

Mortality counts, crude mortality rates (CMRs), and age-adjusted mortality rates (AAMRs) with 95% confidence intervals (CIs) were calculated per 100,000 population for each stratum by year. AAMRs were computed by standardizing death counts to the age distribution of the 2000 U.S. Standard Population to eliminate confounding by differences in age structure across subgroups and time points (11).

Temporal trend fitting was performed using Joinpoint Regression Program version 5.0.1 (National Cancer Institute, Bethesda, MD, USA). A log-linear model was adopted with a maximum of three joinpoints allowed per subgroup; permutation test was used to select optimal inflection points at *α*=0.05. Missing and privacy-suppressed annual observations were assigned zero weight during model fitting. The average annual percent change (AAPC) and segmented annual percent change (APC) with corresponding 95% CIs were estimated to quantify temporal trends. Pairwise *Z*-tests were conducted to compare differences in AAPC values between demographic subgroups; *P* < 0.05 was defined as statistically significant for both APC/AAPC estimates and between-group comparisons. Joinpoint regression was not applied to the Hispanic subgroup for continuous annual trend modeling due to widespread suppressed annual data points. Only full-period aggregate AAPC based on 1999 and 2024 endpoints is reported for this group.

## 3 Results

### 3.1 Overall mortality trends (1999–2024)

From 1999 to 2024, the total number of osteoporosis-related deaths in the United States decreased significantly, from 1,622 deaths in 1999 to 484 in 2024, representing a −70.16% reduction over the 26-year period (Table 1). Consistent with the declining death count, the age-adjusted mortality rate (AAMR) also dropped substantially, from 4.78 (95% CI: 4.54–5.01) in 1999 to 0.92 (95% CI: 0.84–1.00) in 2024. The average annual percent change (AAPC) in AAMR was −6.26 (95% CI: −7.37–−5.14, *P* < 0.05), indicating a steady and statistically significant downward trend in overall mortality attributable to osteoporosis during the study period (Table 1).

### 3.2 Mortality trends by sex

Marked differences in mortality trends were observed between females and males. In 1999, females accounted for 1,466 deaths, while males accounted for 156 deaths. By 2024, female deaths decreased to 416 (−71.62%), and male deaths decreased to 68 (−56.41%). The AAMR in females declined from 6.39 (95% CI: 6.07–6.72) to 1.27 (95% CI: 1.15–1.40), with an AAPC of −6.09 (95% CI: −7.12–−5.04, *P* < 0.05). In males, the AAMR fell from 1.37 (95% CI: 1.15–1.59) to 0.30 (95% CI: 0.23–0.39), with an AAPC of −6.13 (95% CI: −7.77–−4.47, *P* < 0.05). Both sexes exhibited statistically significant declining trends (Figure 1).

**Figure 1:**
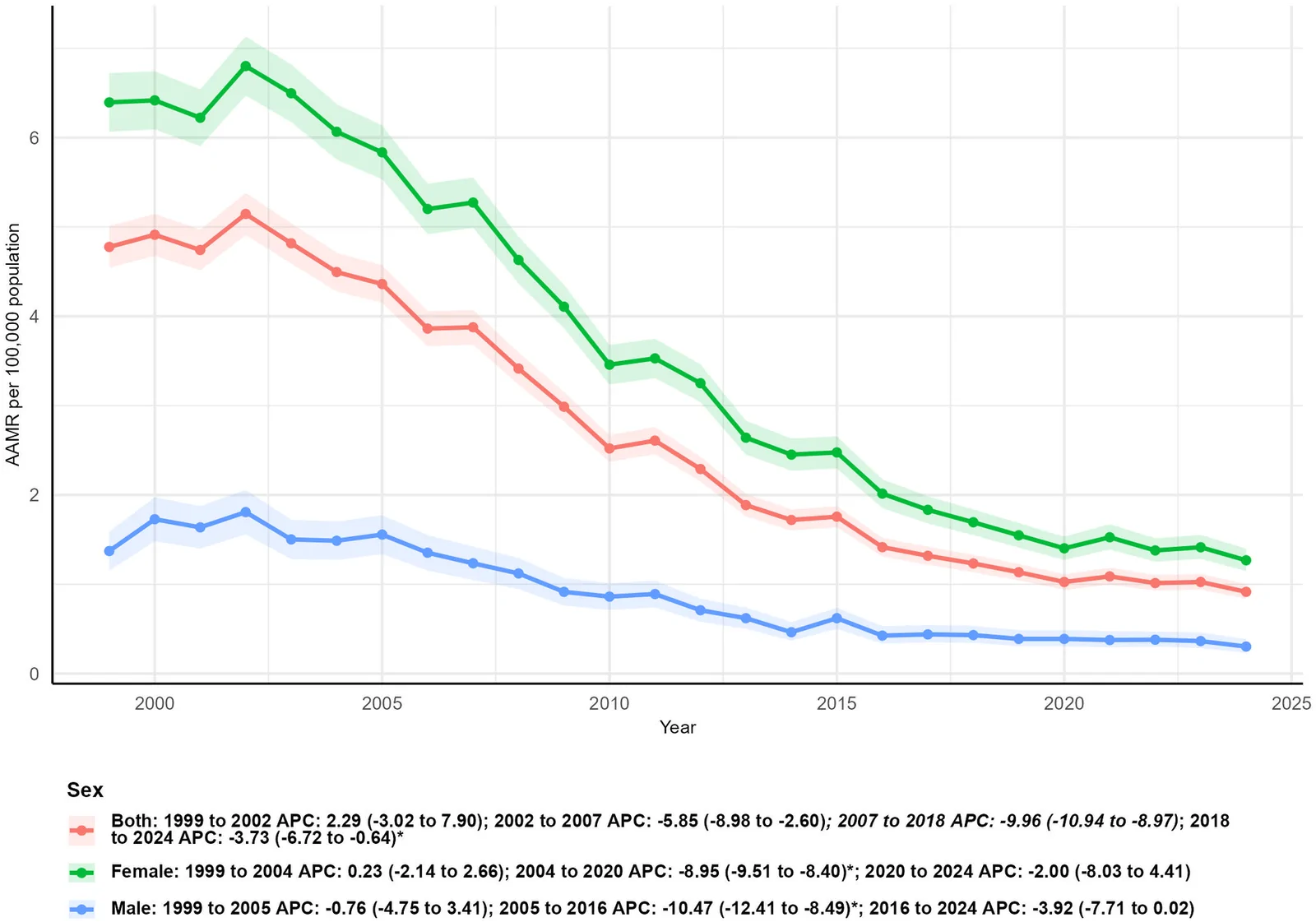
Age-adjusted mortality rates (AAMR) of osteoporosis as the underlying cause of death by sex among U.S. adults aged ≥65 years, 1999–2024 Solid lines: AAMR per 100,000 population; shaded bands: 95% confidence intervals (95% CI). AAMR, age-adjusted mortality rate; APC, annual percentage change; AAPC, average annual percentage change; CI, confidence interval. Asterisk (*) denotes segmented APC statistically different from zero (*P* < 0.05). Groups: Female, Male, Total population.

### 3.3 Mortality trends by census region

All four U.S. Census Regions (Northeast, Midwest, South, West) exhibited significant reductions in osteoporosis-related mortality. The Northeast region showed a −71.14% change in deaths (246–71), with an AAPC of −5.66 (95% CI: −7.16 to −4.13, *P* < 0.05). The Midwest region decreased by −70.76% (513–150), with an AAPC of −5.85 (95% CI: −7.24–−4.44, *P* < 0.05). The South region declined by −68.78% (442–138), with the steepest AAPC of −7.01 (95% CI: −8.73–−5.26, *P* < 0.05). The West region decreased by −70.31% (421–125), with an AAPC of −6.85 (95% CI: −7.88–−5.80, *P* < 0.05). All regional trends were statistically significant (Figure 2).

**Figure 2:**
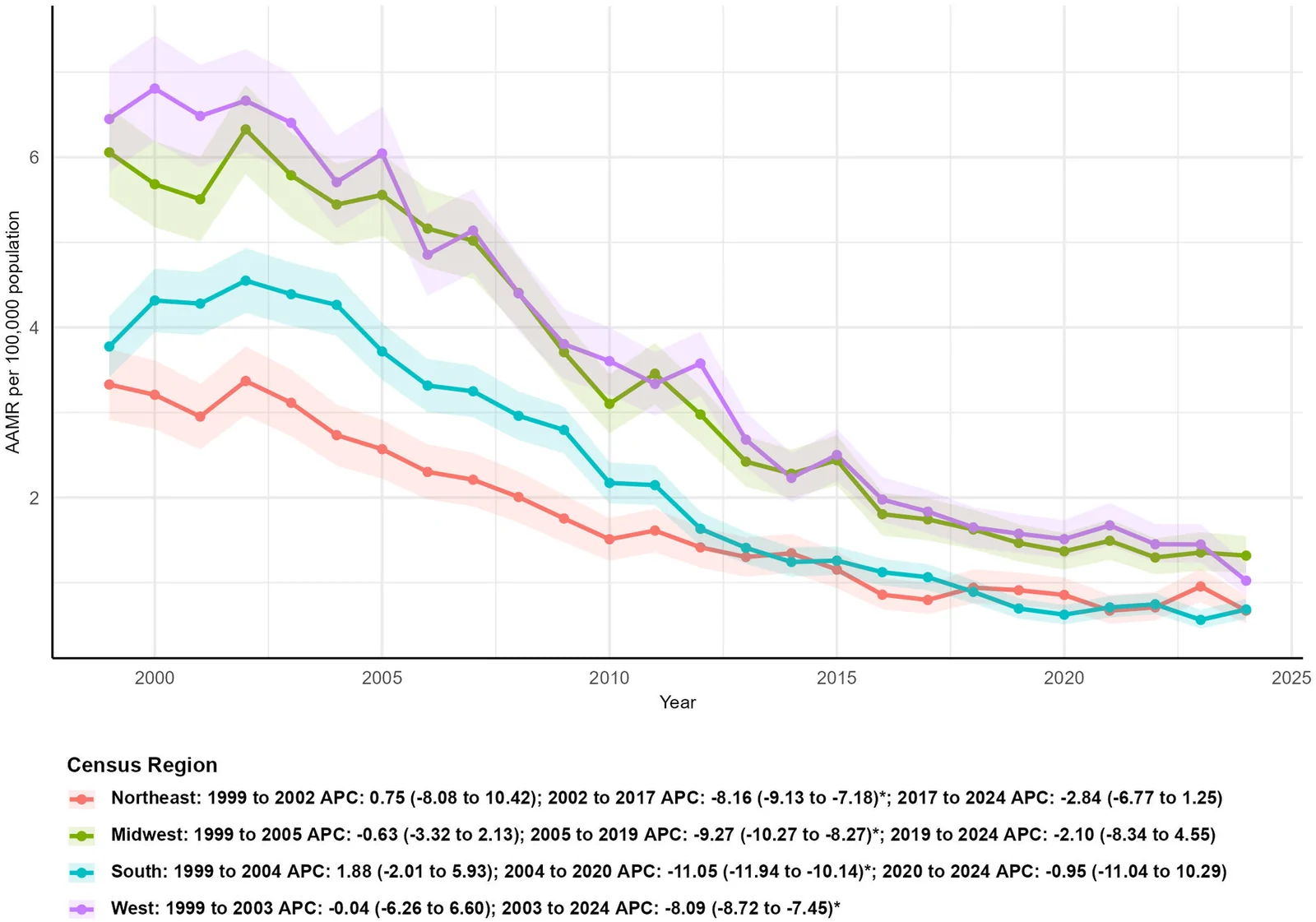
Age-adjusted mortality rates (AAMR) of osteoporosis as the underlying cause of death by U.S. census region among adults aged ≥65 years, 1999–2024 Solid lines: AAMR per 100,000 population; shaded bands: 95% confidence intervals. AAMR, APC, AAPC, CI defined above. * *P* < 0.05 for segmented APC. Groups: Northeast, Midwest, South, West.

### 3.4 Mortality trends by race/ethnicity

Substantial overall reductions in mortality were observed across all three racial/ethnic groups, with complete aggregate metrics summarized in Table 1.

Hispanic group: Total deaths decreased from 28 (1999) to 15 (2024), percent change −46.43%; full-period AAPC = −6.65 (95% CI: −7.78–−5.84, *P* < 0.05). Annual time-series observations were frequently privacy-suppressed due to case counts <10, so continuous annual trend plotting is not feasible for this subgroup.

Non-Hispanic (NH) White group: Deaths declined from 1,548 to 442 (−71.45%), AAPC = −6.01 (95% CI: −6.99–−5.01, *P* < 0.05). Complete unsuppressed annual data support continuous trend visualization.

NH Other group: Deaths decreased from 40 to 27 (−32.50%), AAPC = −5.38 (95% CI: −9.77–−0.77, *P* < 0.05). Complete annual data available for plotting.

All racial/ethnic subgroups demonstrated significant full-period downward trends, while segmented APC results for NH White and NH Other suggested slowing declines or plateauing after 2019. Revised Figure 3 only visualizes NH White and NH Other subgroups with complete continuous annual data, with explicit annotation explaining the exclusion of the Hispanic group due to CDC privacy data suppression (Figure 3).

**Figure 3:**
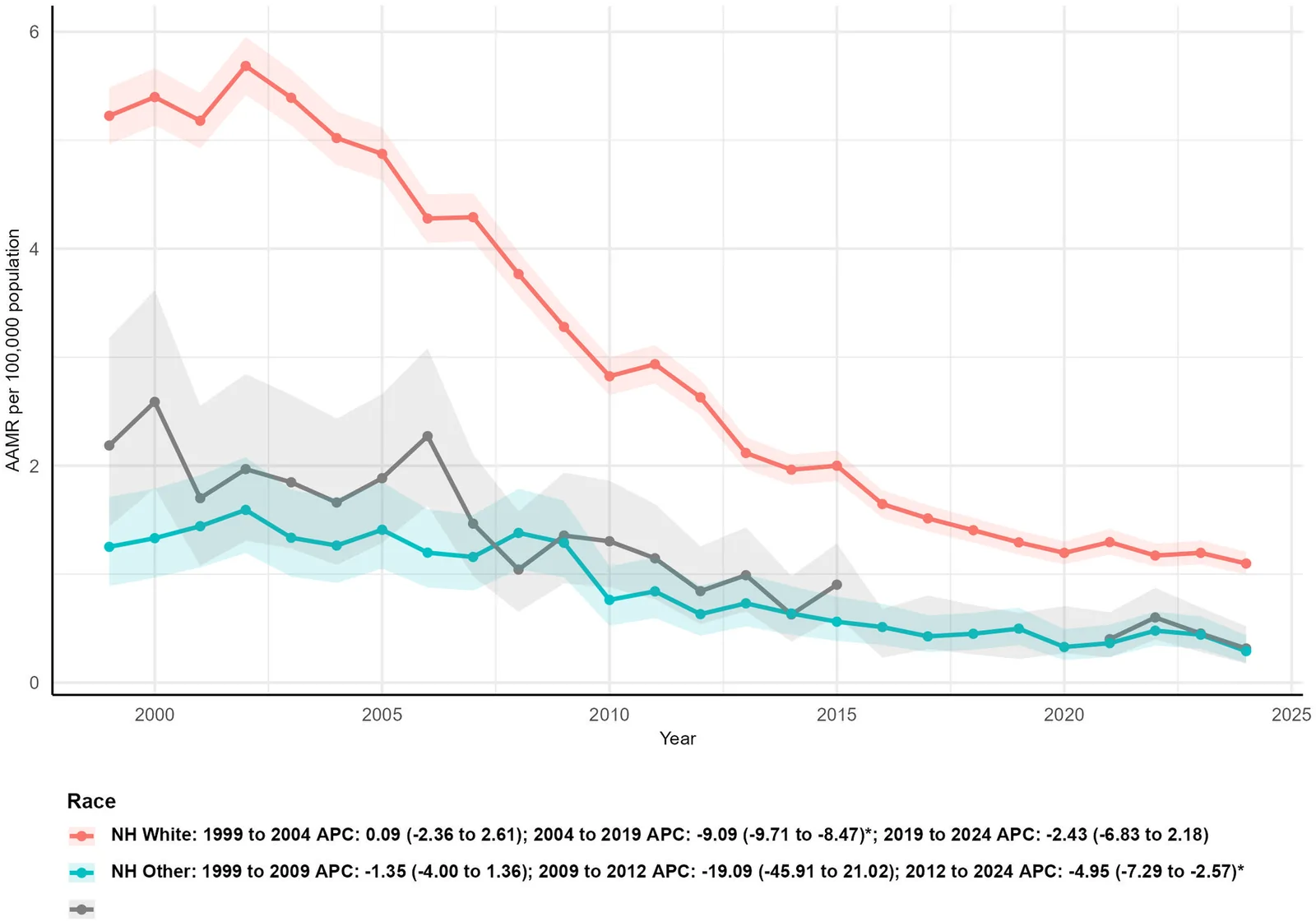
Age-adjusted mortality rates (AAMR) of osteoporosis as the underlying cause of death by race/ethnicity among U.S. adults aged ≥65 years, 1999–2024 Solid lines: AAMR per 100,000 population; shaded bands represent 95% confidence intervals for each subgroup trend line. AAMR, age-adjusted mortality rate; APC, annual percentage change; AAPC, average annual percentage change; CI, confidence interval; NH, non-Hispanic. Asterisk (*) indicates segmented APC estimate statistically different from zero (*P* < 0.05). Plotted subgroups: Non-Hispanic White, Non-Hispanic Other. Critical note on excluded Hispanic subgroup: Annual death counts for Hispanic decedents were frequently <10 across 1999–2024, and CDC WONDER automatically suppresses all cells with counts <10 for privacy protection. Fragmented, discontinuous annual observations cannot generate a reliable continuous trend line for visualization. Complete aggregate cross-sectional mortality metrics and full-period AAPC for Hispanic participants are fully reported in Table 1 and Results Section 3.4.

### 3.5 Mortality trends by urbanization level

Mortality rates were consistently higher in nonmetropolitan areas than in metropolitan areas throughout the restricted 1999–2020 study window for urban-rural stratification. Metropolitan areas showed a −69.46% reduction in deaths (1,251–382), with an AAPC of −7.53 (95% CI: −8.14–−6.91, *P* < 0.05). Nonmetropolitan areas decreased by −72.50% (371–102), with an AAPC of −6.16 (95% CI: −7.15–−5.16, *P* < 0.05). Pairwise comparison confirmed metropolitan subgroups had a significantly faster annual mortality reduction. Both urbanization categories exhibited statistically significant declining trends over 1999–2020; no 2024 urban-rural rate estimates are reported in tables or figures to eliminate time inconsistency (Figure 4).

**Figure 4:**
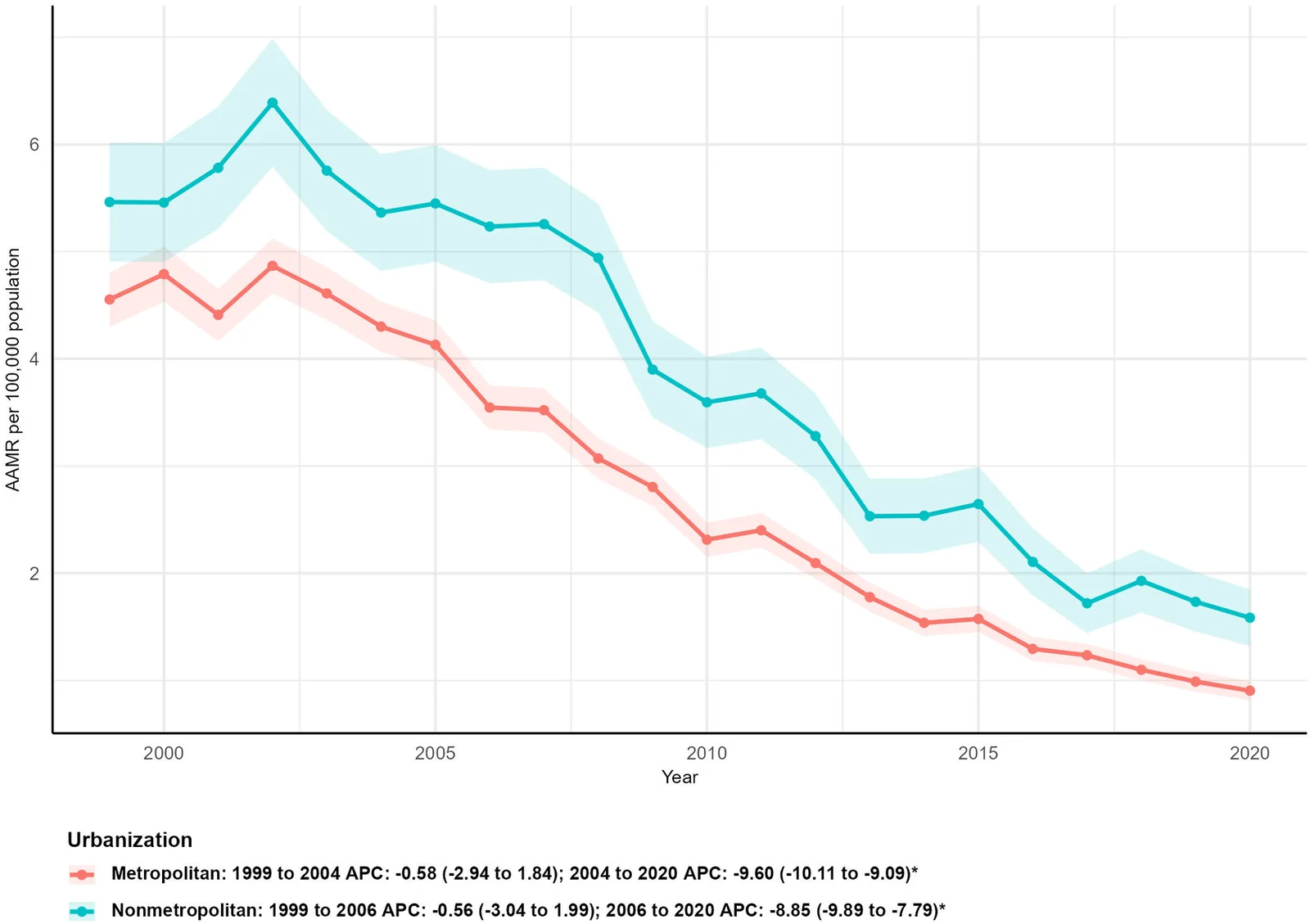
Age-adjusted mortality rates (AAMR) of osteoporosis as the underlying cause of death by urbanization level among U.S. adults aged ≥65 years, 1999–2020 Solid lines: AAMR per 100,000 population; shaded bands = 95% confidence intervals. AAMR, APC, AAPC, CI defined above. * *P* < 0.05 for segmented APC. Groups: Metropolitan, Nonmetropolitan. NCHS 2013 urban-rural classification data are only available through 2020; no 2024 urbanization metrics are plotted or reported.

### 3.6 Mortality trends by age group

Age was strongly associated with mortality where osteoporosis was listed as the underlying cause, with the oldest age group carrying the highest absolute burden. The 65–74 years group showed a −55.71% change in deaths (70–31), with an AAPC of −6.27 (95% CI: −9.87–−2.54, *P* < 0.05). The 75–84 years group declined by −74.30% (428–110), with an AAPC of −7.04 (95% CI: −8.95–−5.10, *P* < 0.05). The 85 + years group decreased by −69.48% (1,124–343), with an AAPC of −6.11 (95% CI: −7.23–−4.98, *P* < 0.05). All elderly age subgroups demonstrated significant reductions in crude mortality rates for the full study period, while segmented APC values indicated plateauing trends after 2017–2019 across all age strata (Figure 5).

**Figure 5:**
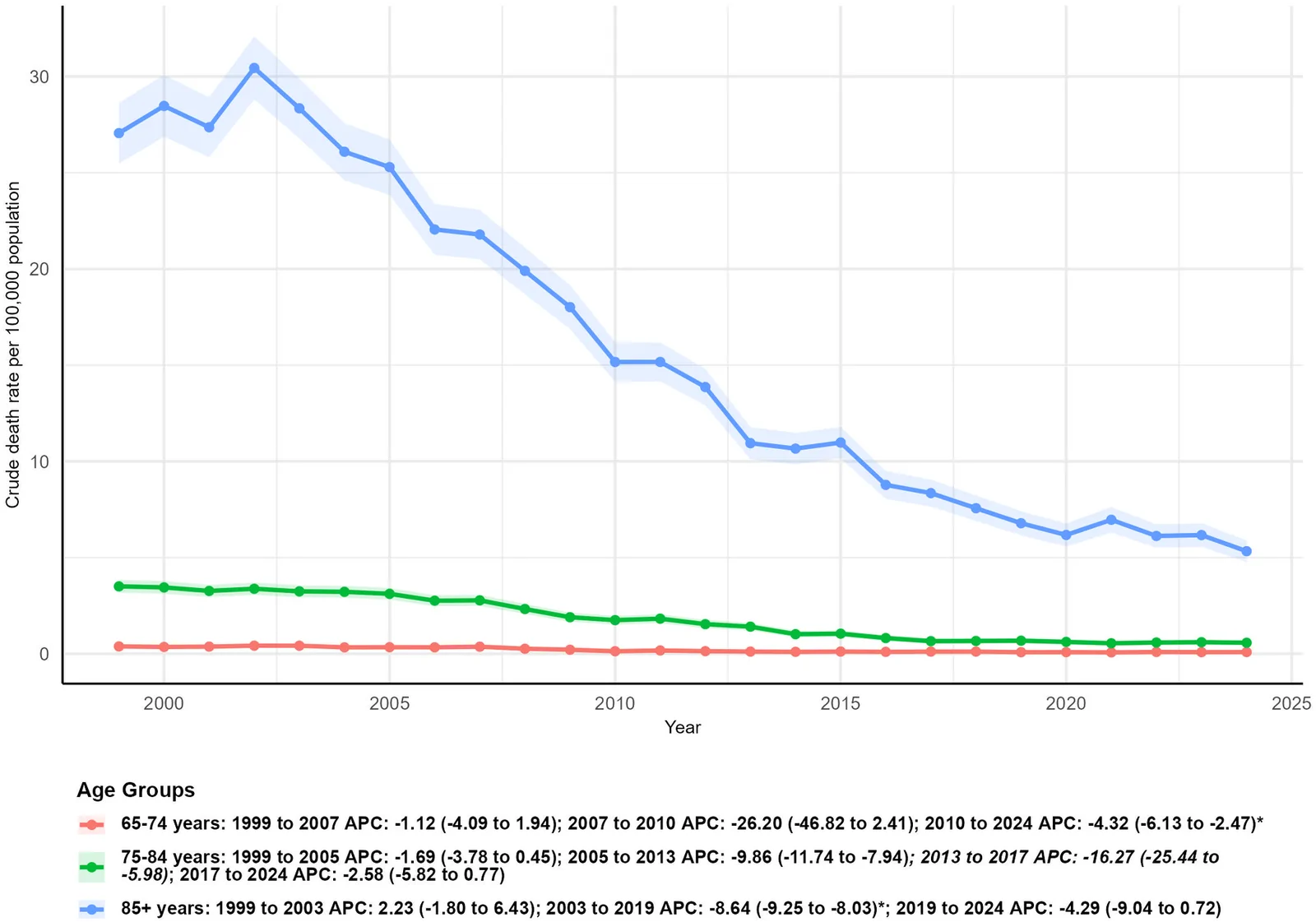
Crude mortality rates (CMR) of osteoporosis as the underlying cause of death by age group among U.S. adults aged ≥65 years, 1999–2024 Solid lines: CMR per 100,000 population; shaded bands = 95% confidence intervals. CMR, crude mortality rate; APC, AAPC, CI defined above. * *P* < 0.05 for segmented APC. Groups: 65–74 years, 75–84 years, ≥85 years.

## 4 Discussion

This study presents a nationally representative analysis of mortality trends with osteoporosis as the underlying cause of death among U.S. adults aged ≥65 years from 1999 to 2024 using the CDC WONDER database. A substantial and statistically significant full-period decrease was observed in total deaths and age-adjusted mortality rates, yet segmented Joinpoint results identified widespread plateauing trends in the most recent years across all stratified subgroups including sex, census region, race/ethnicity, urbanization level, and age group. A key data limitation is persistent privacy suppression of small annual death counts for Hispanic older adults, preventing continuous trend visualization for this subgroup, though aggregate cross-sectional metrics confirm a significant long-term mortality decline for Hispanic participants. These findings provide updated population-level evidence regarding temporal patterns of mortality attributable to osteoporosis when coded as the underlying cause of death.

Females exhibited a higher absolute number of deaths and AAMR compared with males throughout the study period, which is consistent with the well-established epidemiological pattern that females bear a greater disease burden of osteoporosis (12, 13). Despite the higher baseline mortality in females, both sexes demonstrated comparable significant annual downward trends without statistically significant between-group differences, suggesting that overall mortality reductions were broadly similar between males and females. These observations may reflect widespread improvements in bone health–related prevention, screening, and clinical management, although formal causal inference cannot be drawn from this observational ecological study.

All four U.S. census regions showed significant full-period reductions in mortality with osteoporosis as the underlying cause. The South region exhibited the most rapid annual decline (AAPC = −7.01%), followed by the West, Northeast, and Midwest, with formal *Z*-test comparisons verifying faster reductions in Southern and Western subgroups. Regional differences in the magnitude of decline may reflect variations in population structure, healthcare access, public health programs, or geographic distribution of osteoporosis risk factors; however, the present data do not allow confirmation of underlying explanatory factors (14). All regions maintained statistically significant full-period decreasing trends, supporting the nationwide nature of historical mortality reduction, even as recent trends flattened.

Analysis by race/ethnicity demonstrated that Non-Hispanic White individuals accounted for the largest proportion of deaths with osteoporosis as the underlying cause, consistent with their population distribution in the United States. All racial/ethnic groups including Hispanic, Non-Hispanic White, and Non-Hispanic Other showed significant full-period reductions in AAMR. The relatively smaller percent decrease in death counts among Hispanic and NH Other groups should be interpreted cautiously, as these changes may be influenced by demographic shifts, population growth, variations in death certificate coding practices, and CDC privacy suppression of small annual cell counts rather than true differences in mortality risk (15, 16). The inability to plot a continuous annual trend curve for Hispanic participants is an inherent limitation of the CDC WONDER database's privacy rules, not incomplete data collection by our research team. No definitive causal interpretation for intergroup disparities can be drawn from this ecological analysis.

Mortality rates remained consistently higher in nonmetropolitan areas than in metropolitan areas over the 1999–2020 urban-rural analysis window, yet both groups demonstrated significant declines. Metropolitan regions exhibited a significantly more rapid annual reduction (AAPC = −7.53%) than nonmetropolitan regions (AAPC = −6.16%). These observations are consistent with previous studies reporting urban–rural disparities in chronic disease mortality (17, 18). Potential contributors may include healthcare availability, screening uptake, socioeconomic resources, or lifestyle factors; however, such interpretations remain hypothetical and require dedicated individual-level investigation.

Age-stratified results confirmed that individuals aged 85 years and older carried the highest mortality burden linked to osteoporosis as the underlying cause, consistent with the strong age-dependency of osteoporosis (19). All elderly age subgroups (65–74, 75–84, and ≥85 years) showed significant full-period reductions in crude mortality rates. The 75–84 age group showed the largest relative percent decrease in death counts, while the oldest group maintained the highest absolute mortality level. These patterns highlight the persistent vulnerability of the oldest-old population, even as long-term overall mortality rates declined and recent trends plateaued.

## 5 Strengths and limitations

The primary strengths of this study include its long study period (1999–2024), large population-based nationwide sample, use of a validated national mortality database, and comprehensive subgroup stratification by demographic, geographic, and urban-rural characteristics. The use of age-adjusted mortality rates and formal Joinpoint AAPC with pairwise between-group *Z*-tests facilitates standardized interpretation of temporal trends and improves comparability across subgroups. We fully report all available aggregate summary statistics for every racial/ethnic subgroup despite plotting restrictions for small groups.

This study has several limitations. First, the retrospective ecological design limits causal inference and is susceptible to ecological fallacy, as individual-level exposures cannot be linked to mortality outcomes. Second, death certificate data rely on accurate coding of underlying causes, which may vary across regions, years, or clinical settings. Third, data on individual-level confounders such as comorbidity, anti-osteoporosis medication use, smoking, calcium or vitamin D intake, physical activity, and access to bone density testing were not available. Fourth, urbanization classification data were only available up to 2020, restricting all metropolitan/nonmetropolitan trend analyses to 1999–2020 and preventing 2024 urban-rural rate estimates. Fifth, for age-group analyses, crude mortality rates were used instead of AAMR, which may be influenced by shifts in population age structure over time. Sixth, CDC WONDER privacy suppression rules eliminate annual observations with <10 deaths, which fragmented the Hispanic racial/ethnic subgroup time series and prevented continuous trend plotting for this population. Finally, residual confounding from unmeasured societal, healthcare, or environmental factors cannot be excluded.

## 6 Conclusion

Mortality with osteoporosis documented as the underlying cause of death declined significantly over the 26-year period 1999–2024 among U.S. adults aged 65 years and older, with significant overall downward AAPCs identified in all analyzed sex, geographic, racial, and age subgroups. Nevertheless, segmented Joinpoint trend analyses revealed clear plateauing patterns in recent calendar years for most populations, countering the assumption of continuous steady reduction across the full time frame. An important data limitation restricts continuous annual trend visualization for Hispanic older adults due to CDC privacy suppression of small annual case counts, though aggregate cross-sectional metrics confirm significant long-term mortality decline for this group. The heaviest mortality burden persistently fell on female decedents, individuals aged ≥85 years, and residents of nonmetropolitan areas. Formal pairwise statistical comparisons verified more rapid historical mortality declines in Southern U.S. regions and metropolitan zones. This long-term nationwide epidemiological evidence provides updated population-level data to facilitate optimized bone health resource allocation, public health screening programs, and clinical intervention strategies targeting high-risk elderly subgroups in the United States.

## Data Availability

Publicly available datasets were analyzed in this study. These data can be found in the CDC WONDER (Wide-ranging Online Data for Epidemiologic Research) database at https://wonder.cdc.gov/. The specific query criteria, ICD-10 codes, and extraction parameters are detailed in Section 2.2 of the article.
